# Exploring the Preventive Potential of Solubilized Sturgeon Oil on Acute Infection with Respiratory Viruses

**DOI:** 10.3390/md23030112

**Published:** 2025-03-05

**Authors:** Seong Ok Park, Erdenebileg Uyangaa, Yong-Kwang Lee, Suk-Hyun Yun, Minyeong Yu, Hyo Jin Kim, Hye Won Cho, Hee Won Byeon, Chong-Kil Lee, Seong Kug Eo

**Affiliations:** 1 Bio-Safety Research Institute, and Core Facility Center for Zoonosis Research (Core-FCZR), College of Veterinary Medicine, Jeonbuk National University, Iksan 54596, Republic of Korea; pso361313@gmail.com (S.O.P.); ukadoko@gmail.com (E.U.); daesori84@naver.com (S.-H.Y.); 0jymy1004@hanmail.net (M.Y.); khjkhc2000@naver.com (H.J.K.); second8137@naver.com (H.W.C.); h.won0205@gmail.com (H.W.B.); 2 BIO R&D Center, Sturgeon Bio Ltd., Co., Cheongju 28581, Republic of Korea; lyg6600@naver.com (Y.-K.L.); cklee@chungbuk.ac.kr (C.-K.L.)

**Keywords:** solubilized sturgeon oil, acute respiratory viral infection, SARS-CoV-2, influenza A virus, respiratory syncytial virus

## Abstract

Acute respiratory viral infections (ARIs) represent a significant global health challenge, contributing heavily to worldwide morbidity and mortality rates. Recent efforts to combat ARIs have focused on developing nasal spray formulations that effectively target the nasal mucosa. However, challenges such as irritation, discomfort, and safety concerns highlight the need for natural, eco-friendly ingredients. In this study, we evaluated the efficacy of solubilized sturgeon oil (SSO), prepared as an oil-in-water nanoemulsion from Siberian sturgeon, as an eco-friendly preventive nasal spray agent against ARIs. Intranasal pre-treatment with SSO effectively inhibited respiratory infections caused by SARS-CoV-2, influenza A virus (IAV), and respiratory syncytial virus (RSV). Additionally, it suppressed viral replication in both nasal and lung tissues. This antiviral effect was linked to reduced pulmonary inflammation, characterized by decreased infiltration of Ly-6C^+^ monocytes and Ly-6G^+^ neutrophils, along with lower pro-inflammatory cytokine levels. Histopathological analyses confirmed that nasal SSO administration significantly mitigated lung inflammation progression caused by viral infections. Notably, the protective effects of SSO against SARS-CoV-2, IAV, and RSV persisted for at least six hours following nasal application. These findings highlight SSO as a promising eco-friendly and safe candidate for nasal spray formulations, providing a potential frontline defense against ARIs.

## 1. Introduction

Acute respiratory viral infections (ARIs) are a leading cause of mortality and morbidity worldwide, regardless of age or sex [1]. According to the World Health Organization (WHO), these infections remain among the world’s most deadly communicable diseases, second only to COVID-19, and ranked as the fifth leading cause of death in 2021 [2]. Among ARIs, lower respiratory tract infections, such as pneumonia and bronchiolitis, are significant contributors to hospital admissions among young children, particularly in low- and middle-income countries [3]. The global spread of SARS-CoV-2, which began in 2019, underscored the critical importance of international public health measures and highlighted the substantial social and economic costs associated with ARI [4]. Despite ongoing efforts, novel SARS-CoV-2 omicron subvariants, including XBB.1.5 and JN.1, continue to emerge [5]. In the post-COVID-19 era, infections caused by other respiratory viruses, such as influenza A virus (IAV) and respiratory syncytial virus (RSV), have reached unprecedented levels [6,7,8]. This situation emphasizes the urgent need for continued advancements in prevention and therapeutic strategies targeting ARIs.

Although some antiviral therapeutics targeting respiratory viruses, such as SARS-CoV-2 and IAV, have been developed and are currently in clinical use [9,10,11], there remains a critical need for the discovery of new compounds to treat severe illnesses induced by ARIs. Typically, respiratory viruses enter the body through the nasal mucosa via droplets or aerosols and subsequently infect the lower respiratory epithelium [12,13,14]. This infection pathway can ultimately lead to bronchiolar and alveolar involvement, resulting in acute respiratory distress syndrome (ARDS) and other severe conditions [12,13]. Thus, preventing both the initial infection of nasal tissues and the subsequent spread to the lower respiratory tract is considered a key strategy to mitigate the progression of severe diseases such as ARDS. In light of this, significant interest has been directed towards developing nasal spray formulations capable of inhibiting viral replication within the nasal mucosa as a preventive approach against respiratory viral infections [15]. This strategy is particularly beneficial for immunocompromised individuals. Formulations incorporating polymers, such as *iota*-carrageenan and xanthan gum, have been designed to inhibit viral adhesion and replication [16,17,18]. Additionally, a nasal spray called “Pathogen Capture and Neutralizing Spray” (PCAN) has been developed using non-pharmaceutical ingredients [19]. However, the repeated use of such polymers is limited due to their potential to irritate the nasal mucosa and cause discomfort. Other studies have investigated the efficacy of nasal sprays containing astrodrimer sodium, which binds to the SARS-CoV-2 Spike protein [20], as well as low pH-buffered phosphate-buffered saline (PBS) solutions that exploit the virus’s sensitivity to acidic environments [21]. Nevertheless, the irritative effects of low pH levels and the sensation of foreign substances in the nasal cavity have significantly restricted the repeated clinical application of these formulations. These findings highlight the urgent need for the development of eco-friendly nasal sprays that are non-irritating, comfortable, and capable of providing sustained protection against respiratory viral infections without causing discomfort or adverse side effects.

Fatty acids (FAs) are crucial for the structure and function of the immune systems in living organisms. As integral components of cell membranes, they possess physicochemical properties that enable recognition and response to immune stimuli [22,23]. Notably, long-chain polyunsaturated fatty acids (PUFAs), such as eicosapentaenoic acid (EPA), docosahexaenoic acid (DHA), and arachidonic acid, serve as precursors to both anti-inflammatory agents (like EPA) and pro-inflammatory eicosanoids, highlighting their dual regulatory role in inflammation [24]. Furthermore, PUFAs have demonstrated antiviral properties by inhibiting viral replication. For example, glycerol monolaurate (GML) and caprylic acid are effective against African swine fever virus [25,26], while ethyl palmitate (EP), derived from *Sauropus androgynus*, has shown activity against the chikungunya virus [27]. Additionally, DHA and EPA have been found to suppress coronavirus replication by alleviating endoplasmic reticulum (ER) stress [28], and linoleic acid is known to inhibit coronavirus growth through the production of transforming growth factor-beta (TGF-β) [29]. Fish oil, which is abundant in various PUFAs, is recognized for its capacity to mitigate the progression of inflammatory diseases, particularly atopic dermatitis [30]. Clinical studies have shown that water-in-oil emulsions containing linoleic acid can improve skin barrier function [31], while gamma-linolenic acid (GLA) has been effectively used to treat atopic dermatitis without significant side effects [32,33]. Siberian sturgeon (*Acipenser baerii*)—native to rivers like the Ob, Yenisei, Lena, and Kolyma—along with its nutrient-rich caviar, is highly valued for its high DHA and EPA content [34,35]. Sturgeon oil, rich in PUFAs, has demonstrated benefits for conditions such as atopic dermatitis and alopecia, potentially through the modulation of gut microbiota [36,37]. Despite the eco-friendly profile and antiviral properties of sturgeon oil, there has been little research into its potential application in nasal spray formulations for the prevention of respiratory viral infections.

Here, we examined the efficacy of solubilized sturgeon oil (SSO) as a key component in nasal spray formulations designed to protect against respiratory viral infections. SSO is an oil-in-water formulation produced via the nanoemulsification and distillation of sturgeon oil [36,38]. The primary advantage of incorporating sturgeon oil into an oil-in-water microemulsion lies in its superior absorption compared to the oil in its native form. Its water-solubility also facilitates easy integration into nasal spray solutions, reducing the sensation of foreign matter and minimizing irritation upon application. Our results revealed that nasal administration of SSO significantly reduced the mortality and morbidity associated with infections by SARS-CoV-2, IAV, and RSV. Additionally, SSO nasal treatment was found to decrease viral loads and inflammatory responses in both nasal and lung tissues. These results suggest that SSO could be a promising candidate for developing non-irritating and safe nasal sprays as an effective preventive strategy against ARIs.

## 2. Results

### 2.1. SSO Pre-Treatment Mitigates Lung Inflammation with Reduced Viral Burden After SARS-CoV-2 Infection

#### 2.1.1. SSO Pre-Treatment Inhibits SARS-CoV-2 Replication and Ameliorates Morbidity in Hamster Infection Model

**Figure 1:**
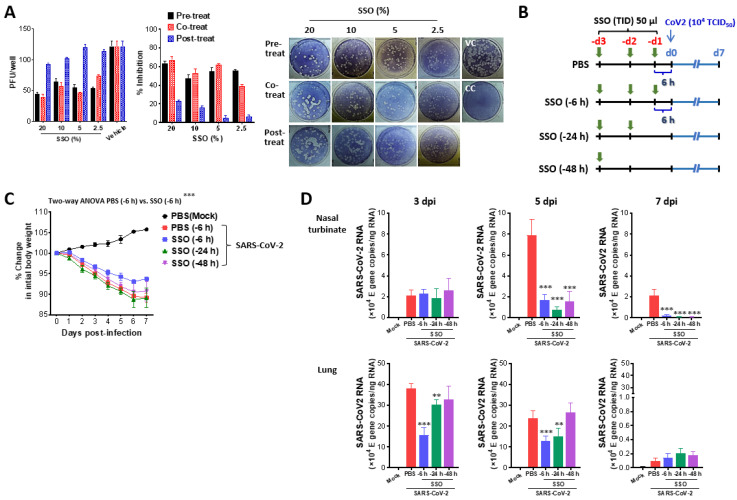
SSO pre-treatment inhibits SARS-CoV-2 replication and ameliorates morbidity in hamster infection model. (**A**) Inhibition of SARS-CoV-2 production by pre- and co-treatment of SSO. Vero E6 cells plated in a 24-well plate were pretreated with SSO, treated simultaneously with SSO during SARS-CoV-2 infection, or treated with SSO after virus infection. SARS-CoV-2 proliferation was assessed based on the number of plaques formed. Representative plaque images were obtained from Vero E6 cells treated with SSO. (**B**) Experimental scheme. Syrian hamsters were pretreated with SSO one time a day for 3 days. Pre-administration of SSO was completed 6, 24, and 48 h before SARS-CoV-2 infection, and SSO-pre-treated hamsters were monitored for changes in body weight for 7 days after virus infection. (**C**) Changes in body weight of SSO-pre-treated hamsters after SARS-CoV-2 infection. Changes in body weight of SSO-pre-treated hamsters were daily monitored for 7 days after SARS-CoV-2 infection. (**D**) SARS-CoV-2 burden in the lung tissues of SSO-pre-treated hamsters. Viral burden in the lung tissues of SSO-pre-treated hamsters was determined by E gene-targeted real-time qRT-PCR using total RNA extracted from tissues at 3, 5, and 7 dpi. The viral RNA load was expressed by SARS-CoV-2 RNA copy number per nanogram of total RNA. The graphs indicate the mean ± SEM of each group (*n* = 5), and results are representative of one out of at least two individual experiments with five hamsters per group. The body weight data were statistically analyzed using a two-way ANOVA. Statistical significance is indicated as * *p* < 0.05, ** *p* < 0.01, *** *p* < 0.001, compared to the PBS-treated group, using a two-tailed unpaired *t*-test.

To investigate the inhibitory effect of SSO on SARS-CoV-2 replication, the SARS-CoV-2 Wuhan strain was used to infect Vero E6 cells as host cells. The experiment included three groups: a post-treatment group, where SSO was administered 1 h after viral infection; a co-treatment group, where SSO was administered simultaneously with the virus; and a pre-treatment group, where Vero E6 cells were pre-treated with SSO 30 min prior to viral infection. The results indicated that administering SSO after SARS-CoV-2 infection did not inhibit viral replication (Figure 1A). In contrast, when SSO was applied concurrently with the virus or as a pre-treatment, the replication of SARS-CoV-2 was suppressed in a dose-dependent manner. Notably, pre-treatment with SSO prior to SARS-CoV-2 infection resulted in more pronounced inhibition of viral replication compared to the co-treatment group. Specifically, a 5% SSO pre-treatment achieved approximately 60% suppression of viral replication compared to the untreated group.

Given the in vitro inhibitory effect of SSO pre-treatment on SARS-CoV-2 replication, we assessed the efficacy of SSO pre-treatment in preventing SARS-CoV-2 infection using a hamster infection model. Hamsters were divided into four groups based on the timing of intranasal SSO administration. The control group received intranasal PBS three times daily, starting 3 days before infection and continuing until 6 h before SARS-CoV-2 exposure. The second group received intranasal SSO following the same schedule as the control group. The third group received SSO for 2 days, with the final dose administered 24 h before infection, and the fourth group received SSO for 1 day 48 h prior to infection (Figure 1B). In this model, SARS-CoV-2 infection typically causes body weight loss in hamsters, followed by gradual weight recovery beginning around 6–7 days post-infection [39]. Accordingly, body weight changes were monitored until day 7 post-infection. The PBS-treated control group exhibited the most significant weight loss. No significant differences in body weight loss were observed between the groups treated with SSO until 24 or 48 h before infection and the PBS-treated group (Figure 1C). However, the group receiving SSO up to 6 h prior to infection showed a significantly slower rate of body weight loss compared to the control group, suggesting that SSO administration closer to the time of infection can mitigate SARS-CoV-2-induced morbidity. To further investigate the reduced body weight loss observed in the group treated with SSO 6 h before infection, viral loads in the nasal turbinate and lung tissues were measured on days 3, 5, and 7 post-infection. On day 3, the viral load in the nasal turbinate tissues did not differ significantly between the SSO-treated and control groups. However, by days 5 and 7, the viral load in the SSO-treated groups was substantially lower than that of the PBS-treated group (Figure 1D). This reduction in nasal turbinate viral load was observed not only in the group treated with SSO 6 h before infection but also in the groups treated 24 and 48 h prior. In lung tissues, SSO administration reduced the viral load as early as day 3 post-infection, with the group treated 6 h before infection exhibiting the most pronounced reduction compared to the PBS-treated group. This trend persisted through day 5. However, by day 7, viral loads in all groups, including the PBS-treated group, had decreased to low levels, with no significant differences among them. In conclusion, pre-treatment with SSO up to 6 h before SARS-CoV-2 infection effectively mitigates SARS-CoV-2 infection-induced morbidity. This protective effect is likely due to the ability of SSO to inhibit the spread of the virus to lung tissue, thereby limiting extensive viral replication in the lungs.

#### 2.1.2. Attenuated Lung Inflammation in SARS-CoV-2-Infected Hamster After SSO Pre-Treatment

**Figure 2:**
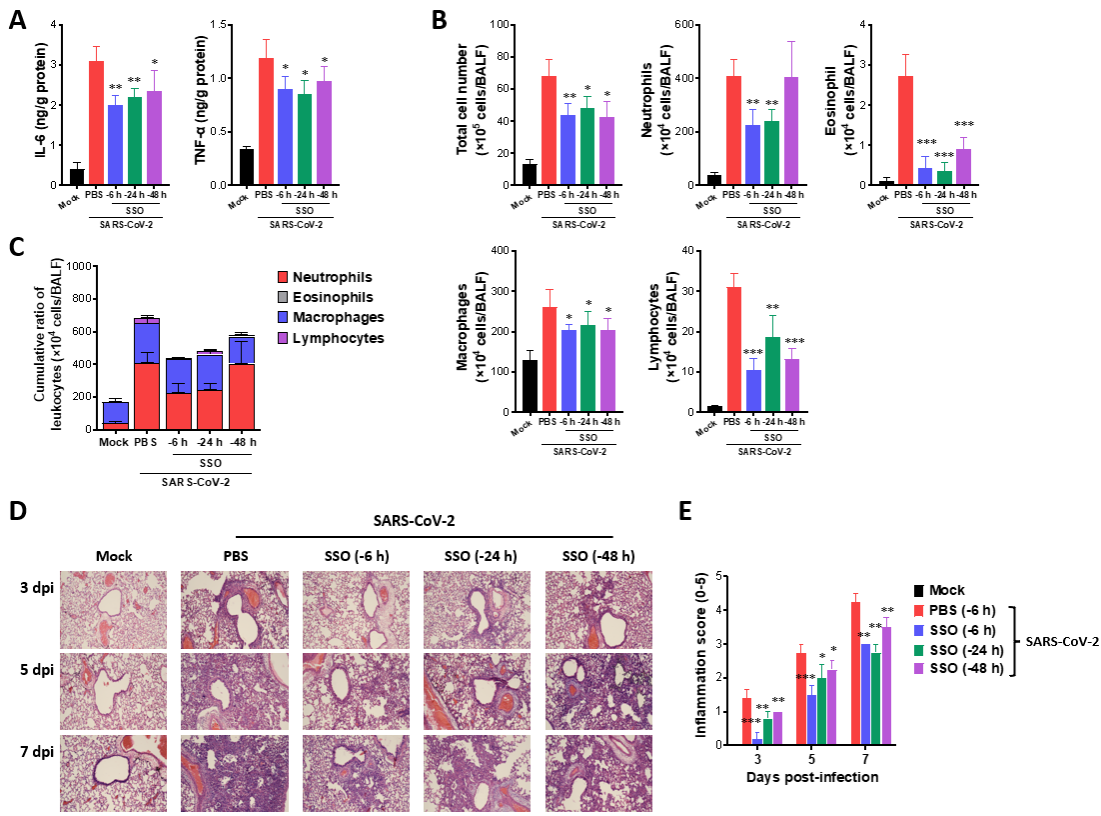
SSO pre-treatment attenuates lung inflammation in SARS-CoV-2-infected hamsters. (**A**) Secreted levels of cytokines in BALF fluid of SSO-pre-treated hamsters. The production of IL-6 and TNF-α was measured by ELISA at 3 days after SARS-CoV-2 infection using BALF fluid harvested from SSO-pre-treated hamsters. (**B**) The number of BALF leukocyte subpopulations in SSO-pre-treated hamsters. (**C**) Cumulative cell number of BALF leukocyte subpopulations in BALF. The leukocytes in BALF were assessed by cytospinning and subsequent Wright–Giemsa staining 3 days after SARS-CoV-2 infection. (**D**) Histopathological pictures of lung tissue derived from SSO-pre-treated-hamsters after SARS-CoV-2 infection. Representative H&E-stained lung sections derived from SSO-pre-treated hamsters were examined at 3, 5, and 7 dpi. Images are representative of sections (200×) from at least 5 hamsters. Represented photomicrographs show inflamed perivascular and peribronchial areas. (**E**) Quantitative analyses of lung inflammation. Inflammation was blind scored 3, 5, and 7 days after SARS-CoV-2 infection. The graphs indicate the mean ± SEM of each group (*n* = 5), and results are representative of one out of at least two individual experiments with five hamsters per group. Statistical significance is indicated as * *p* < 0.05, ** *p* < 0.01, *** *p* < 0.001, compared to the PBS-treated group, using a two-tailed unpaired *t*-test.

To better understand how SSO pre-treatment mitigates SARS-CoV-2-induced morbidity and inhibits viral spread to lung tissue, inflammatory cytokine levels in the lungs were measured. As shown in Figure 2A, SSO pre-treatment reduced the production of inflammatory cytokines IL-6 and TNF-α on day 3 post-infection, with the lowest levels observed in the group that received intranasal SSO 6 h prior to infection. In addition, an analysis of inflammatory immune cells infiltrating BALF on day 3 post-infection demonstrated that SSO pre-treatment significantly decreased immune cell infiltration (Figure 2B). Notably, the group treated with SSO 6 h before infection exhibited marked suppression of the total number of inflammatory cells, including reduced neutrophil and eosinophil infiltration. A cumulative analysis confirmed that the cumulative number of infiltrating immune subpopulations in the six-hour pre-treatment group was lower than in the groups treated 24 or 48 h prior to infection (Figure 2C). These results suggest that intranasal administration of SSO before SARS-CoV-2 infection effectively suppresses lung inflammation. Specifically, administering SSO closer to the time of infection further attenuates inflammation markers induced by the virus. To investigate the impact of SSO on virus-induced pulmonary inflammation in more detail, a histopathological analysis of lung tissue was performed. The results showed that lung inflammation was significantly reduced in the group treated 6 h before infection compared to the groups treated 24 or 48 h prior (Figure 2D). Additionally, hamsters pre-treated with SSO exhibited less inflammatory cell infiltration around the bronchi and blood vessels. Quantification of lung inflammation revealed that the group treated 6 h before infection had the lowest inflammation score (Figure 2E). In summary, these findings indicate that intranasal SSO administration prior to SARS-CoV-2 infection suppresses the progression of virus-induced pulmonary inflammation. Furthermore, administering SSO closer to the time of infection enhances its effectiveness in reducing the severity of lung inflammation.

### 2.2. Pre-Treatment of SSO Attenuates Mortality and Morbidity Caused by Infection with IAV

#### 2.2.1. Attenuation of Mortality and Morbidity by Pre-Treatment of SSO at Least 6 H Prior to IAV Infection

**Figure 3:**
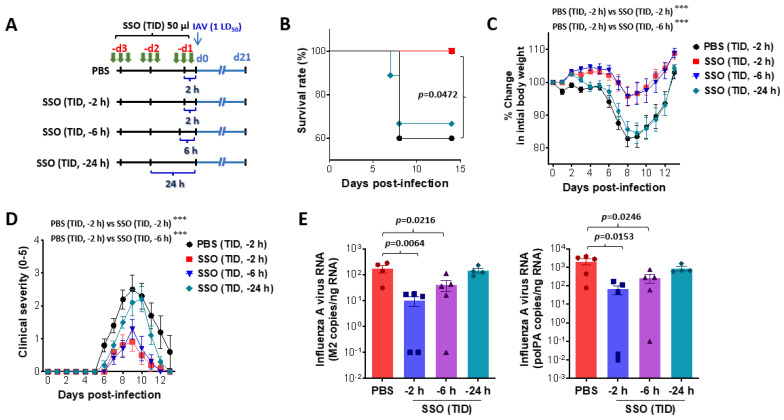
SSO pre-treatment mitigates mortality and morbidity in IAV-infected mice by reduction of viral burden. (**A**) Experimental scheme. Mice were pretreated with SSO three times a day for 3 days. Pre-administration of SSO was completed 2, 6, and 24 h before IAV infection, and SSO-pre-treated mic were monitored for mortality and morbidity for 21 days after virus infection. (**B**–**D**) Attenuated mortality and morbidity of SSO-pre-treated mice to IAV infection. SSO-pre-treated mice were monitored daily for survival rate (**B**), change of body weight (**C**), and clinical score (**D**) after IAV infection. (**E**) Viral burden in the lung tissues of SSO-pre-treated mice following IAV infection. Viral burden in the lung tissues was determined by M2 and polPA gene-targeted real-time qRT-PCR using total RNA extracted from tissues. The viral RNA load was expressed by IAV RNA copy number per nanogram of total RNA (*n* = 4–5). Each symbol represents the level in an individual mouse; the bar indicates the mean ± SEM of each group. Data in graphs denote the mean ± SEM. Results are representative of one out of at least two individual experiments with four to five mice per group. The body weight and clinical severity data were statistically analyzed using a two-way ANOVA. Statistical significance is indicated as * *p* < 0.05, ** *p* < 0.01, *** *p* < 0.001, compared to the PBS-treated group, using a two-tailed unpaired *t*-test.

IAV infection is a significant respiratory pathogen, comparable to SARS-CoV-2, and is recognized as a potential cause of pandemic infections [40]. Given that SSO pre-treatment reduces morbidity, viral load, and pulmonary inflammation induced by SARS-CoV-2, we investigated whether it could similarly mitigate IAV-induced pulmonary disease. To evaluate this, animals were divided into several groups. The control group received intranasal PBS three times daily, starting 3 days before IAV infection and continuing until 2 h before viral exposure. The other groups received intranasal SSO following different schedules: one group was administered SSO three times daily for 3 days, ending 2 h before infection; another group followed the same regimen, but treatment ended 6 h before infection; and the final group received SSO three times daily for 2 days, ending 24 h before infection (Figure 3A). IAV-infected animals typically develop clinical symptoms between days 5 and 6 post-infection, with some cases resulting in mortality. Therefore, mortality was monitored for 14 days post-infection. In the control group, 40% of the infected animals succumbed to IAV infection. In contrast, no mortality was observed in the groups treated with SSO up to 2 or 6 h before infection (Figure 3B). However, the group treated with SSO 24 h prior to infection exhibited approximately 30% mortality, indicating that intranasal SSO administration at least 6 h before infection is necessary to achieve protective effects. Body weight changes were also monitored following IAV infection. Animals treated with SSO 2 and 6 h before infection showed minimal body weight loss (Figure 3C). In contrast, the group treated with SSO 24 h before IAV infection experienced rapid weight loss similar to the PBS-treated control group. Clinical symptoms were further quantified using behavioral scoring. The groups treated with SSO up to 2 or 6 h before IAV infection had low clinical scores, while the group treated 24 h prior to infection exhibited high clinical scores, comparable to those of the control group (Figure 3D). Given the observed reduction in mortality and morbidity with SSO pre-treatment, we measured viral load in lung tissue post-infection. The results showed that the closer SSO administration occurred to the time of infection, the lower the viral load detected in the lung tissue (Figure 3E). Therefore, the lowest viral load was observed in animals treated with SSO up to 2 h prior to infection. In summary, these findings indicate that intranasal administration of SSO prior to IAV infection inhibits viral replication in lung tissue, thereby reducing virus-induced mortality and morbidity. Furthermore, the protective effect of SSO is enhanced when administered closer to the time of infection.

#### 2.2.2. SSO Pre-Treatment Ameliorates Lung Inflammation with Reduced Production of Cytokine Secretion After IAV Infection

**Figure 4:**
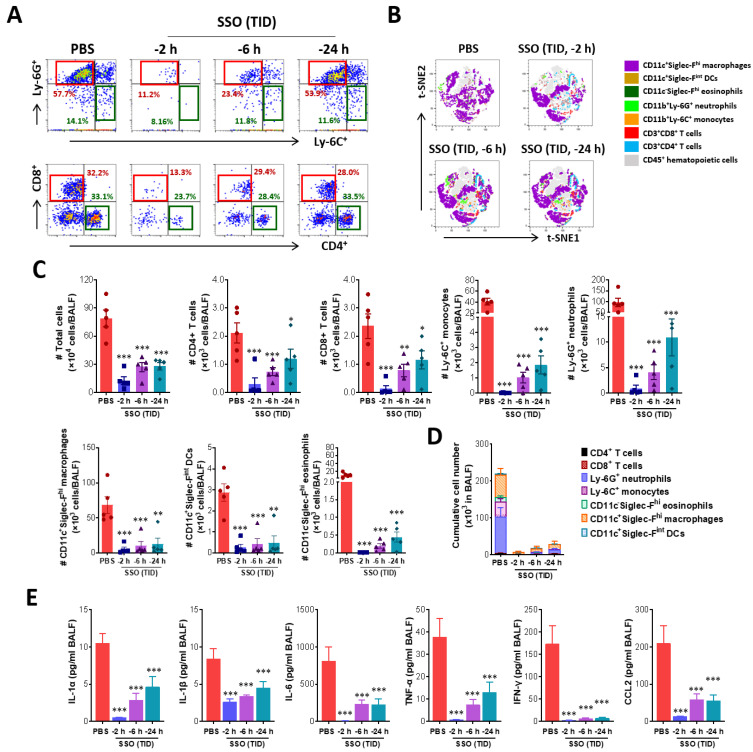

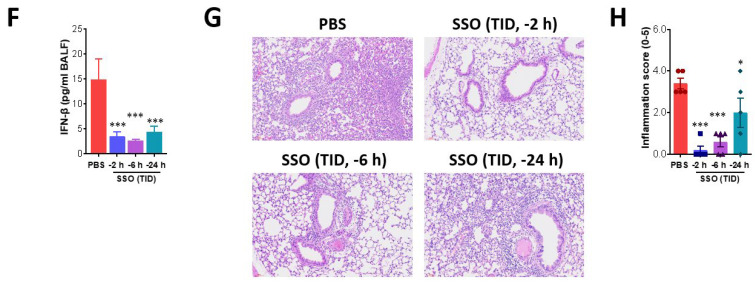
Amelioration of lung inflammation in IAV-infected mice following SSO pre-treatment. (**A**) The frequency of infiltrated Ly–6C^+^ and Ly–6G^+^ neutrophils in BALF. BALF was harvested from SSO-pre-treated mice 5 dpi and used for the analysis of infiltrated Ly–6C^+^ monocytes and Ly–6G^+^ neutrophils using flow cytometer. The values in dot-plots represent the average percentage of the indicated cell population after gating on CD11b^+^ cells. (**B**) *t*-SNE maps describing the local probability of lymphoid and myeloid cells in BALF. Representative *t*-SNE maps shows the local probability of lymphoid (CD4^+^, CD8^+^ T cells) and myeloid cell subpopulations (Ly–6C^+^ monocytes, Ly–6G^+^ neutrophil, CD11c^−^Siglec-F^hi^ eosinophils, CD11c^+^Siglec-F^hi^ macrophages, CD11c^+^Siglec-F^int^ dendritic cells) at 5 dpi (**C**) Total number of lymphoid and myeloid subpopulations in BALF. (**D**) Cumulative cell number of lymphoid and myeloid subpopulations in BALF. BALF leukocytes harvested from SSO-pre-treated mice were employed in 6-color flow cytometry analysis to examine lymphoid and myeloid cell subpopulations at 5 dpi. (**E**) Cytokine secretion in BALF. BALF fluid was harvested from SSO-pre-treated mice at 5 dpi and used for CBA to examine the levels of secreted cytokines. (**F**) The production of type I IFN (IFN-β) in BALF. The production of IFN-β was measured by ELISA at 5 dpi using BALF fluid harvested from SSO-pre-treated mice. (**G**) Histopathological pictures of lung tissue derived from SSO-pre-treated-mice after IAV infection. Representative H&E-stained lung sections derived from SSO-pre-treated mice were examined at 5 dpi. Images are representative of sections (200×) from at least 5 mice. Represented photomicrographs show inflamed perivascular and peribronchial areas. (**H**) Quantitative analyses of lung inflammation. Inflammation was blind scored 5 days after IAV infection. Each symbol represents the level in an individual mouse; the bar indicates the mean ± SEM of each group. Data in graphs denote the mean ± SEM. Results are representative of one out of at least two individual experiments with four to five mice per group. Statistical significance is indicated as * *p* < 0.05, ** *p* < 0.01, *** *p* < 0.001, compared to the PBS-treated group, using a two-tailed unpaired *t*-test.

Given that SSO pre-treatment suppresses IAV-induced mortality, clinical symptoms, and viral replication, we next examined its effect on pulmonary inflammation following IAV infection. On day 5 post-infection, we analyzed the infiltration of inflammatory immune cells, specifically Ly-6C⁺ monocytes and Ly-6G⁺ neutrophils, in the BALF. The results indicated that animals pre-treated with SSO closer to the time of infection exhibited lower levels of these immune cells in the BALF (Figure 4A). Similar trends were observed for CD4⁺ and CD8⁺ T cells, with the group treated with SSO up to 2 h before infection showing the lowest levels of all these immune cells. In contrast, animals treated with SSO up to 24 h before infection exhibited levels of Ly-6C⁺ monocytes, Ly-6G⁺ neutrophils, CD4⁺ T cells, and CD8⁺ T cells comparable to those of the PBS-treated control group. Additionally, a *t*-distributed Stochastic Neighbor Embedding (*t*-SNE) map illustrating the distribution of myeloid and lymphoid cells in the BALF revealed that CD11c⁺Siglec-F^hi^ macrophages constituted the majority of infiltrating immune cells (Figure 4B). However, the frequency of CD11c⁺Siglec-F^hi^ macrophages was lower in the SSO-treated groups compared to the control group. SSO pre-treatment significantly inhibited the infiltration of Ly-6C⁺ monocytes and Ly-6G⁺ neutrophils, with the most substantial reduction observed when SSO was administered closer to the time of infection. Quantification of total myeloid and lymphoid cell counts in the BALF further confirmed that SSO pre-treatment markedly suppressed immune cell infiltration (Figure 4C). Notably, animals treated with SSO 2 h before infection exhibited the lowest levels of both myeloid and lymphoid cell infiltration. Similarly, cumulative analysis showed that the 2 h SSO pre-treatment group had the lowest overall infiltration levels (Figure 4D). The group treated with SSO up to 6 h before IAV infection also demonstrated significant suppression of immune cell infiltration, though to a lesser extent than the 2 h group.

To further explore the effect of SSO pre-treatment on pulmonary inflammation, we measured the levels of inflammatory cytokines secreted into the BALF. The results indicated that animals receiving SSO closer to the time of infection exhibited reduced cytokine secretion (Figure 4E). Notably, the group treated with SSO up to 2 h before infection had the lowest levels of inflammatory cytokines, including CCL2, a chemokine that promotes immune cell infiltration [41]. Type I interferons (IFNs; IFN-α/β) are crucial for inhibiting viral replication [42]. Measurement of IFN-β levels in the BALF showed that the 2 h SSO pre-treatment group had the lowest IFN-β production, consistent with the reduced secretion of other inflammatory cytokines (Figure 4F). These findings suggest that type I IFN production correlates with the extent of viral replication. The effect of SSO pre-treatment on pulmonary inflammation was further evaluated through histopathological examination. As expected, SSO pre-treatment significantly suppressed lung inflammation induced by IAV infection compared to the PBS-treated control group (Figure 4G). Specifically, animals treated with SSO up to 2 h before infection showed no observable infiltration of inflammatory cells around the bronchi and blood vessels, indicating that SSO pre-treatment was most effective when administered closer to the time of infection. To quantify the impact of SSO pre-treatment on pulmonary inflammation, inflammation scores were assigned based on histopathological findings. The results confirmed that animals treated closer to the time of infection exhibited lower inflammation scores, with the 2 h SSO pre-treatment group showing the most pronounced reduction (Figure 4H). Taken together, these findings demonstrate that SSO pre-treatment significantly suppresses the progression of pulmonary inflammation induced by IAV infection. Moreover, the closer SSO is administered to the time of infection, the greater its inhibitory effect on lung inflammation, highlighting its potential as a preventive intervention for IAV-induced pulmonary disease.

### 2.3. Attenuated Infection of RSV by Pre-Treatment of SSO

**Figure 5:**
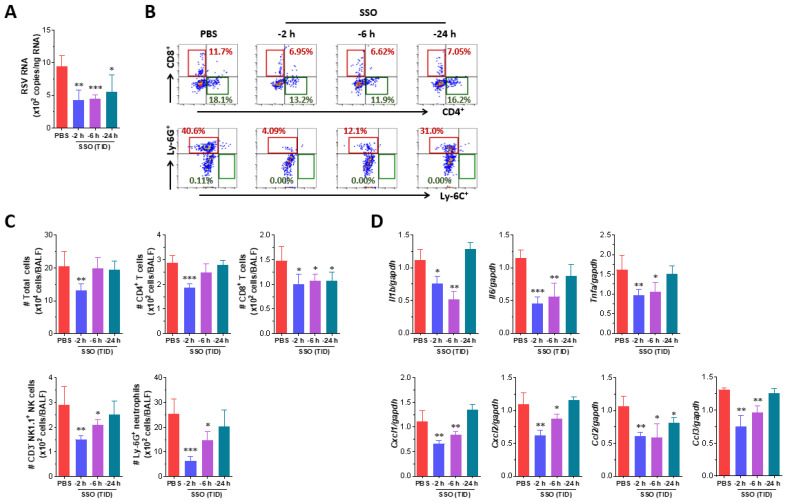
SSO pre-treatment attenuates RSV replication and inflammatory responses in the lung. (**A**) Viral burden in the lung tissues of SSO-pre-treated mice following RSV infection. Viral burden in the lung tissues was determined by F gene-targeted real-time qRT-PCR using total RNA extracted from tissues 3 days following RSV infection. The viral RNA load was expressed by RSV RNA copy number per nanogram of total RNA. (**B**) The frequency of infiltrated immune cells in BALF. BALF was harvested from SSO-pre-treated mice 3 dpi and used for the analysis of infiltrated immune cells including CD4^+^, CD8^+^ T cells, Ly-6C^+^ monocytes and Ly-6G^+^ neutrophils using flow cytometer. The values in dot-plots represent the average percentage of the indicated cell population after gating on CD3^+^ and CD11b^+^ cells for T cells and myeloid cells, respectively. (**C**) Total number of lymphoid and myeloid subpopulations in BALF following RSV infection. (**D**) Expression levels of inflammatory cytokines in the lung tissues of SSO-pre-treated mice following RSV infection. The expression of inflammatory cytokine mRNAs was determined by real-time qRT-PCR using total RNA extracted from tissues 3 dpi and normalized to housekeeping GAPDH expression. The graphs indicate the mean ± SEM of each group (*n* = 5–6), and results are representative of one out of at least two individual experiments. Statistical significance is indicated as * *p* < 0.05, ** *p* < 0.01, *** *p* < 0.001, compared to the PBS-treated group, using a two-tailed unpaired *t*-test.

RSV is a respiratory pathogen known to cause severe pulmonary diseases, particularly in infants and older adults [43,44]. In the post-COVID-19 era, concerns over RSV spread have increased following the global outbreak of SARS-CoV-2 [45]. Given that SSO pre-treatment suppresses SARS-CoV-2- and IAV-induced pulmonary inflammation, we investigated its efficacy in mitigating RSV-induced pulmonary inflammation. The experimental design for SSO pre-treatment followed the same structure as that used for IAV infection, and viral loads in lung tissue were measured following RSV infection. The results showed that RSV levels in the lungs were lower in animals pre-treated with SSO compared to the control group treated with PBS up to 2 h before infection (Figure 5A). Notably, animals receiving SSO closer to the time of infection exhibited further reductions in viral load. We next analyzed the infiltration of inflammatory immune cells, including Ly-6C⁺ monocytes, Ly-6G⁺ neutrophils, CD4⁺ T cells, and CD8⁺ T cells, into the BALF following RSV infection. While RSV infection induced minimal infiltration of Ly-6C⁺ monocytes, Ly-6G⁺ neutrophil infiltration increased significantly in the PBS-treated control group (Figure 5B). In contrast, SSO pre-treatment effectively suppressed Ly-6G⁺ neutrophil infiltration, with the most substantial reduction observed in the groups treated with SSO up to 2 or 6 h prior to RSV infection. Animals treated up to 24 h before infection exhibited only modest suppression of neutrophil infiltration. Similarly, animals pre-treated with SSO up to 2 or 6 h before RSV infection showed reduced infiltration of CD4⁺ and CD8⁺ T cells. Quantification of total immune cell counts in the BALF confirmed that SSO pre-treatment significantly inhibited inflammatory immune cell infiltration, with the strongest effect observed when SSO was administered closer to the time of infection (Figure 5C). Specifically, the 2-h and 6-h SSO pre-treatment groups contained a significantly lower total number of Ly-6G⁺ neutrophils in the BALF compared to the PBS-treated control group. Finally, we assessed the progression of pulmonary inflammation by analyzing the expression of inflammatory cytokines in lung tissue following RSV infection. As expected, animals treated with SSO up to 2 or 6 h before RSV infection exhibited lower levels of inflammatory cytokine expression compared to the control group (Figure 5D). In contrast, animals treated up to 24 h before infection did not show a significant reduction in cytokine expression. These findings indicate that intranasal administration of SSO at least 6 h before RSV infection suppresses viral replication and mitigates pulmonary inflammation. SSO pre-treatment, therefore, holds potential as an effective preventive strategy against RSV-induced pulmonary disease.

## 3. Discussion

In this study, we demonstrate that SSO is a promising eco-friendly and safe component for nasal spray formulations targeting respiratory viral infections. Various nasal spray ingredients have been developed for this purpose. Some formulations, such as high-molecular-weight polymers like *iota*-carrageenan and astodrimer sodium [16,17,19,20], form a protective film on the nasal mucosa, blocking viral attachment. While effective, polymer-based sprays can cause discomfort due to film formation, limiting their widespread use. Consequently, research has focused on developing safer, eco-friendly alternatives. Promising candidates include Dimocarpus longan extract (P80 natural essence) [46] and nitric oxide derivatives [47], both of which have demonstrated efficacy against SARS-CoV-2 and IAV. Low-pH PBS-based sprays have also been explored [21], though irritation remains a concern. In this context, we propose SSO as a novel nasal spray ingredient. Processed into a water-soluble oil-in-water nanoemulsion, SSO is non-irritating and suitable for repeated use [34,35,36]. Traditionally consumed as a health food, sturgeon oil is considered safe, and its high PUFA content may contribute to the inhibition of respiratory virus replication [25,26,28,29]. This study confirms that SSO effectively suppresses infections caused by SARS-CoV-2, IAV, and RSV. The surfactant properties of PUFAs further suggest that SSO may exert potent inhibitory effects on envelope-containing respiratory viruses, making it a strong candidate for nasal spray development.

Fish oil, rich in PUFAs, has demonstrated therapeutic benefits in various conditions, including atopic dermatitis suppression [30] and inflammation reduction [31]. Sturgeon oil has also been linked to improvements in alopecia by modulating gut microbiota [37]. PUFAs, essential dietary components that cannot be synthesized by the human body, play critical roles in viral inhibition. Structurally, they are long-chain carbon molecules classified as ω-6 or ω-3 fatty acids. Linoleic acid, the primary ω-6 PUFA, is metabolized into bioactive compounds such as GLA and arachidonic acid, while ALA, an ω-3 PUFA, is converted into EPA and DHA [48]. PUFAs have been shown to inhibit viral replication, with DHA and EPA reducing ER stress to block coronavirus replication [28]. Linoleic acid and arachidonic acid also suppress the replication of human coronavirus and MERS-CoV [49], while GML exhibits strong antiviral activity [25]. Additionally, 18-hydroxy eicosapentaenoic acid (HEPE), an ω-3 fatty acid metabolized by gut microbiota, enhances IFN-λ production, suppressing pneumonia progression in IAV infection [50]. Although the potential influence of the herbal extract included in SSO on respiratory viral inhibition cannot be entirely ruled out, we propose that PUFAs in SSO play a key role in inhibiting SARS-CoV-2, IAV, and RSV replication upon nasal entry. This inhibition leads to reduced viral loads in nasal and lung tissues, decreased inflammatory cytokine production, and lower inflammatory cell infiltration in BALF, ultimately mitigating lung inflammation. Furthermore, SSO has been shown to promote filaggrin production, a structural protein essential for antimicrobial defense, skin hydration, and barrier maintenance [36,51,52]. It also upregulates tight junction proteins such as claudin-1, occludin, and ZO-1 [36], which may help protect nasal mucosal cells from viral injury and prevent virus spread to the lower respiratory tract. Future studies should further investigate the precise mechanisms underlying SSO’s antiviral effects in nasal tissues.

In the post-COVID-19 era, respiratory infections continue to rise, posing significant health risks and economic burdens [45]. To address ARIs, researchers have focused on nasal sprays, with PCAN-based formulations offering approximately eight hours of protection and *iota*-carrageenan-based sprays providing longer efficacy [16,17,18,19]. However, the film-forming properties of *iota*-carrageenan can cause irritation, limiting its usability. The present study found that SSO effectively prevents respiratory viral infections for at least six hours. Based on our results, an SSO-containing nasal spray administered approximately four times daily could provide continuous protection. Considering typical daily routines, applying the spray three to four times during waking hours may be an effective strategy for infection prevention. While SSO appears to be a promising ingredient for nasal spray formulations, the characteristic odor of fish oil may cause discomfort for users. To address this issue, future research should focus on developing alternative formulations to mitigate the distinctive scent of SSO in nasal sprays. By overcoming this limitation, an SSO-based nasal spray formulation offers a promising, eco-friendly, and safe solution as a primary defense against ARIs, particularly as respiratory infections continue to rise in the post-pandemic era.

## 4. Materials and Methods

### 4.1. Ethics Statement

All animal experiments described in the present study were conducted at Jeonbuk National University according to the guidelines set by the Institutional Animal Care and Use Committees (IACUC) of Jeonbuk National University and were pre-approved by the Ethical Committee for Animal Experiments of Jeonbuk National University (approval number: CBNU-2019-00204, NON2022-039-002). The animal research protocol in this study followed the guidelines set up by the nationally recognized Korea Association for Laboratory Animal Sciences (KALAS). Biosafety experiments were approved by the Institutional Biosafety Committee (IBC) of Jeonbuk National University (no.: JBNU 2020-11-003-002) and were performed in a biosafety cabinet at the BL3 and ABL3 facilities in Core Facility Center for Zoonosis Research (Core-FCZR), Jeonbuk National University.

### 4.2. Animals, Cells, and Viruses

Wild-type (WT) C57BL/6 (H-2^b^, 6-week-old female) mice were purchased from SAMTAKO (Osan, Republic of Korea) or Damool Science (Daejeon, Republic of Korea), and golden Syrian hamsters (*Mesocricetus auratus*) were obtained from Central Lab Animal Inc. (Seoul, Republic of Korea) or Saeronbio Inc. (Uiwang, Republic of Korea). The SARS-CoV-2 Wuhan prototype (NCCP 43326) was kindly provided by the National Culture Collection for Pathogens (NCCP), National Institute of Infectious Diseases (NIID), and National Institute of Health (NIH) (O-song, Republic of Korea). This virus was handled as an infectious and hazardous agent under BL3 conditions in a dedicated ABL3 facility. The SARS-CoV-2 virus was propagated in Vero E6 cells (ATCC CRL-1586) maintained with DMEM supplemented with 2% FBS, penicillin (100 U/mL), and streptomycin (100 U/mL). Supernatants exhibiting cytopathic effects were harvested, and viral concentrations were determined using a plaque assay. Influenza A/PR/08/34 (H1N1) virus (IAV), propagated via inoculation into the chorioallantoic membrane of embryonated eggs, was generously provided by Professor Jeong-Ki Kim at the College of Pharmacy, Republic of Korea University (Sejong, Republic of Korea). Human respiratory syncytial virus (RSV) strain A2 (ATCC, VR-1540) was obtained from the American Type Culture Collection (ATCC) (Manassas, VA, USA) and propagated in HEp-2 cells (ATCC, CCL-23) derived from human laryngeal carcinoma. The propagated RSV was titrated using a focus-forming assay with goat anti-RSV polyclonal antibody (Millipore, Temecula, CA, USA) and stored at −80 °C until use.

### 4.3. Antibodies and Reagents

The following mAbs were obtained from BD Biosciences (San Diego, CA, USA), Biolegend (San Diego, CA, USA), and Invitrogen (Waltham, MA, USA) for FACS analysis and other experiments: FITC-labeled anti-CD45 (30-F11), CD3 (145-2C11); PE-labeled anti-CD11b (M1/70), CD11c (N418), CD4 (RMA4-5); PerCP/Cyanine5.5-labeled anti-Ly-6C (HK1.4), Siglec-F (E50-2440); PE/Cy7-labeled anti-NK1.1 (PK136); APC-labeled anti-Ly-6G (1A8) and CD8 (53–6.7).

### 4.4. Preparation and Analysis of SSO

The methods for SSO preparation are presented in detail in a patent registered with the Korean Intellectual Property Office [38], as previously described elsewhere [36]. The oil layer was extracted from a boiling-water extract of the Siberian sturgeon (*Acipenser baerii*), diluted with purified water (1:100), and sprayed using a high-pressure syringe pump to form a nanoemulsion. This nanoemulsion was combined with an herbal extract (200:1) made from wild ginseng and other medicinal plants, aged in a sterile container for 10 days at 19–23 °C, and distilled to produce SSO. The Korea Quality Testing Institute analyzed the total lipid content (0.53 ± 0.06%) using the Soxhlet-petroleum ether extraction method and the fatty acid (FA) composition via gas chromatography-mass spectrometry, as presented elsewhere [36].

### 4.5. Acute Infection Models of Respiratory Viruses

SSOs were administered intranasally three times a day for three days, up to 2, 6, and 24 h before the viral challenge. Briefly, the animals were carefully handled, and 50 µL of SSO was placed at the tip of each mouse’s nose to facilitate inhalation through natural breathing. For the SARS-CoV-2 challenge, Syrian hamsters were administered the SARS-CoV-2 Wuhan prototype at a dose of 1 × 10^6^ PFU via intranasal instillation. Infected hamsters were euthanized on days 3, 5, and 7 post-infection to analyze viral replication, cytokine levels, and histopathology. To induce IAV infection, C57BL/6 mice were anesthetized and infected intranasally with a dose equivalent to 1 LD_50_. Infected mice were euthanized on day 5 post-infection to analyze viral replication, cytokine levels, and histopathology. For RSV infection, C57BL/6 mice were anesthetized and infected with 5 × 10^6^ FFU via the intranasal route. The mice were euthanized on day 4 post-infection to assess viral replication. All infected animals were monitored daily for body weight and clinical symptoms, which were systematically recorded throughout the infection period.

### 4.6. Collection and Analysis of Bronchoalveolar Lavage Fluid (BALF)

Infected animals were anesthetized via intramuscular injection of zolazepam (Zoletil 50; Virbac, Carros, France) prior to thoracotomy. The trachea was exposed and punctured with scissors, allowing the insertion of a 21-gauge winged infusion set connected to a syringe containing 0.6 mL of PBS. The lungs were gently flushed with the PBS solution, and this process was repeated twice more, each time using 1.0 mL of PBS. The first BALF sample was centrifuged at 1000 rpm for 5 min, and the supernatant was used for cytokine secretion analysis. The cells obtained from the first BALF were pooled with those from the second and third BALF samples for leukocyte analysis. To assess differential leukocytes in the BALF, cell suspensions (1 × 10^5^ cells/200 μL) were prepared using a Cytospin system (Thermo Scientific, Waltham, MA, USA). Slides were then air-dried and stained with Wright–Giemsa stain solution (Muto Pure Chemicals Ltd., Tokyo, Japan). After staining, eosinophils, neutrophils, macrophages, and lymphocytes were identified based on cell morphology and blindly counted using light microscopy (Olympus Corp., Tokyo, Japan).

### 4.7. Quantitative Real-Time RT-PCR for Determination of Viral Burden and Cytokine Expression

The viral load and cytokine expression in nasal turbinate and lung tissues were quantified using real-time qRT-PCR. Tissues were homogenized with Wizol™ reagent (WizbioSolution, Seongnam, Republic of Korea) and grinding beads in a tissue homogenizer (Precellys Evolution, Bertin, France), followed by total RNA extraction from the supernatant. RNA concentrations were measured using a NanoDrop2000 spectrophotometer (Thermo Scientific). One microgram of total RNA was reverse-transcribed into cDNA using the WizScript™ cDNA synthesis kit (WizbioSolution), and the cDNA was amplified by real-time qPCR with WizPure™ qPCR Master-UDG (WizbioSolution) on a BioRad CFX Connect Real-Time PCR System (BioRad, Hercules, CA, USA). The viral load was calculated as RNA copy numbers using standard curves generated with specific primers and probes (Table 1). Cytokine expression levels were quantified by amplification with specific primers and normalized to GAPDH expression for comparative analysis. All data were analyzed using Bio-Rad CFX Manager software (version 2.1). Control reactions without template DNA were included in each assay, and all experiments were conducted in duplicate, with product authenticity confirmed by melting curve analysis.

### 4.8. Determination of Secreted Cytokine Proteins

Cytokine expression in BALF was measured using a cytokine bead array (CBA) according to the manufacturer’s protocol (BioLegend LEGENDplex, Available online: https://www.biolegend.com/en-us/legendplex (accessed on 11 December 2024)). Cytokine levels were quantified as concentrations per mL of BALF, while protein levels of various cytokines and chemokines in lung tissues were assessed using the LEGENDplex Mouse Inflammation Panel (BioLegend, 740446), normalized to protein content determined by the Bradford method. Additionally, IL-6, TNF-α, and IFN-β levels in BALF and lung tissues were quantified using sandwich ELISA following the manufacturer’s protocols (eBioscience, BD Bioscience, Invitrogen). For ELISA, Nunc MaxiSorp 96-well plates were coated with capture antibodies, followed by incubation with samples, detection using biotinylated antibodies and streptavidin-HRP, and color development with TMB substrate. Optical density (OD) values at 450 nm were measured using an ELISA reader (Molecular Devices, San Jose, CA, USA). Cytokine concentrations were calculated using SoftMax Pro 3.4 software, with reference to standard cytokine protein concentrations.

### 4.9. Flow Cytometric Analysis

Inflammatory immune cells infiltrated in BALF were analyzed by staining BALF leukocytes with an antibody cocktail, and the fluorescently labeled cells were acquired using a flow cytometer (Gallios, Beckman Coulter, Brea, CA, USA). The focus of the analysis was on the infiltrating inflammatory immune cells, including CD3^+^CD4^+^ and CD3^+^CD8^+^ T cells, CD3^−^NK1.1^+^ NK cells, CD11b^+^Ly-6C^+^ monocytes, CD11b^+^Ly-6G^+^ neutrophils, CD11c^+^Siglec-F^int^ dendritic cells (DCs), CD11c^+^Siglec-F^hi^ macrophages, and CD11c^−^Siglec-F^hi^ eosinophils using FlowJo software (version 10.6; Ashland, OR, USA; Cat No. 664187).

### 4.10. Histopathological Examinations

Histopathological examinations were performed on left lung lobes perfused with 10% neutral buffered formalin. Lung tissues were paraffin-embedded, sectioned (10 μm), and stained with hematoxylin and eosin (H&E). Slides were scanned and analyzed using a slide scanner (Motic Digital Pathology, Kowloon, Hong Kong). Lung inflammation and goblet cell hyperplasia were graded with a semiquantitative scoring system. Inflammation was evaluated on a 5-point scale: 0 (standard) to 5 (high cell influx, significant pathology). Five fields per section were counted, and mean scores from 5 to 6 mice per group were calculated. Analyses were conducted blindly, with slides presented randomly.

### 4.11. Statistical Analysis

All data are expressed as the average ± standard error of the mean (SEM). For the ex vivo experiments and immune cell analyses, statistically significant differences between groups were analyzed using unpaired two-tailed student’s *t*-testing. For multiple comparisons, statistical significance was determined using one-way or two-way analysis of variance (ANOVA) with repeated measures, followed by Bonferroni post hoc tests. The statistical significance of the in vivo cytokine gene expression was evaluated using unpaired two-tailed Student’s *t*-testing. A *p*-value ≤ 0.05 was considered significant. All data were analyzed using GraphPadPrism 9.0.0 software (GraphPad Software, Inc., La Jolla, CA, USA).

## Figures and Tables

**Table 1:** Specific primers and probes used for real-time PCR analysis.

Gene Name	Primer Sequence (5′–3′)	Gene Bank ID
*IL-1β*	F: AAG TGA TAT TCT CCA TGA GCT TTG T R: TTC TTC TTT GGG TAT TGC TTG G	NM_008361.4

*IL-6*	F: AAC GAT GAT GCA CTT GCA GA R: GAG CAT TGG AAA TTG GGG TA	NM_0.31168.2

*TNF-a*	F: CGT CGT AGC AAA CCA CCA AG R: TTG AAG AGA ACC TGG GAG TA	NM_013693.3

*Cxcl1*	F: CGC TGC TGC TGC TGG CCA CCA R: GGC TAT GAC TTC GGT TTG GGT GCA	NM_008176.3

*Cxcl2*	F: ATC CAGAGC TTG AGT GTGACG R: AAG GCA AAC TTT TTG ACC GCC	NM_009140.2

*Ccl2*	F: AAA AAC CTG GAT CGG AAC CAA R: CGG GTC AAC TTC ACA TTC AAA G	NM_011333.3

*Ccl3*	F: CCA AGT CTT CTC AGC GCC AT R: GAA TCT TCC GGC TGT AGG AG	NM_011337.2

*GAPDG*	F: AAC GAC CCC TTC ATT GAC R: TCC ACG ACA TAC TCA GCA C	NM_001289726.1

SARS-CoV-2	F: ACAGGTACGTTAATAGTTAATAGCGT	OX489524.1
	R: ATATTGCAGCAGTACGCACACA	
	Probe: [FAM]ACACTAGCCATCCTTAC[BHQ1]	
IAV	^a^ F: CGGTCCAAATTCCTGCTGA	CY121306.1
	R: CATTGGGTTCCTTCCATCCA	
	Probe: [HEX]CCAAGTCATGAAGGAGA[BHQ1]	
IAV	^b^ F: CTTCTAACCGAGGTCGAAACGTA	OK022520.1
	R: GGTGACAGGATTGGTCTTGTCTTTA	
	Probe: [FAM]TCAGGCCCCCTCAAAGC[BHQ1]	
RSV-A	F: TTGGATCTGCAATCGCCA	M22643.1
	R: CTTTTGATCTTGTTCACTTCTCCTTCT	
	Probe: [HEX]TGGCACTGCTGTATCTA[BHQ1]	

^a^ Polymerase PA, ^b^ Matrix protein.

## Data Availability

The data supporting the conclusions of this article are included within the article. Original slides, photographs, and FACS dot-plots are retained. All reagents used in this study are available from scientific supply companies, except for SSO. The datasets used and analyzed during the current study are available from the corresponding author on reasonable request.
